# Control of *in vivo* ictogenesis via endogenous synaptic pathways

**DOI:** 10.1038/s41598-017-01450-8

**Published:** 2017-05-02

**Authors:** Hiram Luna-Munguia, Phillip Starski, Wu Chen, Stephen Gliske, William C. Stacey

**Affiliations:** 1 Department of Neurology, University of Michigan, Ann Arbor, MI 48109 USA; 2 Department of Biomedical Engineering, University of Michigan, Ann Arbor, MI 48109 USA

## Abstract

The random nature of seizures poses difficult challenges for epilepsy research. There is great need for a reliable method to control the pathway to seizure onset, which would allow investigation of the mechanisms of ictogenesis and optimization of treatments. Our hypothesis is that increased random afferent synaptic activity (i.e. synaptic noise) within the epileptic focus is one endogenous method of ictogenesis. Building upon previous theoretical and *in vitro* work showing that synaptic noise can induce seizures, we developed a novel *in vivo* model of ictogenesis. By increasing the excitability of afferent connections to the hippocampus, we control the risk of temporal lobe seizures during a specific time period. The afferent synaptic activity in the hippocampus was modulated by focal microinjections of potassium chloride into the *nucleus reuniens*, during which the risk of seizure occurrence increased substantially. The induced seizures were qualitatively and quantitatively indistinguishable from spontaneous ones. This model thus allows direct control of the temporal lobe seizure threshold via endogenous pathways, providing a novel tool in which to investigate the mechanisms and biomarkers of ictogenesis, test for seizure threshold, and rapidly tune antiseizure treatments.

## Introduction

Epilepsy is one of the world’s most prevalent and debilitating diseases, affecting 1% of the population and severely affecting quality of life for patients and caregivers. Decades of research have improved our understanding of some of the physiological changes that lead to epilepsy and have led to several antiseizure medications. Recent technological advances have begun to examine seizures in more detail, uncovering some of their dynamical properties^1,2^, and describing behavior of individual cells to help understand how those dynamics are formed^3,4^. Yet despite this research, we still have a very limited understanding of the basic mechanisms of ictogenesis—the process by which an individual seizure begins. Without this understanding, epilepsy research depends upon waiting for seizures to occur randomly, an uncontrolled and time-consuming process. Developing a rigorous, physiological method to control seizure threshold would open many new avenues of research.

There are currently two basic strategies to account for the random nature of seizures in epilepsy research. The first requires monitoring over long periods of time to capture multiple seizures. Most epilepsy research follows this method, as it assures the seizures are naturally-occurring and spontaneous. However, this method is very inefficient and affords no control over seizure onset time nor the processes that induce seizures. The second strategy is to induce seizures artificially using either proconvulsant drugs^5^ or kindling with electrical stimulation^6^. These induction methods allow direct control of seizure onset time, but unfortunately they have limited applicability to endogenous seizures because they function via different mechanisms and are known to behave differently than spontaneous seizures^6–8^. While these strategies have been the foundation for decades of epilepsy research, neither provides direct experimental control of endogenous seizures or seizure threshold, which greatly limits the ability to investigate the mechanisms and biomarkers of ictogenesis.

In this work, we present a novel method of controlling ictogenesis in an *in vivo* model of temporal lobe epilepsy in rats. Our hypothesis is that a seizure focus can be triggered via random afferent synaptic activity, or “synaptic noise”, without any direct manipulation of the neurons within the focus itself. This hypothesis is based upon the physical properties of noise within a coupled neural network, which under certain conditions can cause the network to activate, synchronize, and generate coherent oscillations. This phenomenon, known as stochastic coherence or coherence resonance, was first identified in simulated neural networks^9–13^, and later shown to be likely within the brain due to the vast number of inputs and noise^14–18^. However, it has been challenging to design experiments to test this phenomenon^19^. There are several methods to modulate synaptic noise—e.g. mechanoreceptors^20^, AC currents^21^, DC currents^22^, and visual stimuli^23^ —but they have not been used to induce seizures. However, one recent experiment triggered seizure-like activity by controlling afferent synaptic noise within an *in vitro* model of intact mouse septo-hippocampal formation^24^. That experiment used a dual chamber in which the bath for the septum could be isolated from the hippocampal bath solution, while maintaining the synaptic connectivity. Potassium chloride (KCl) added to the septum chamber slightly depolarized cells within the septum, which increased their random firing and triggered seizures downstream in the hippocampus. That experiment effectively proved that synaptic noise can induce seizures *in vitro*.

The goal of the current work is to utilize a similar strategy to modulate hippocampal seizure onset *in vivo*, i.e. a model that allows control of seizure risk. Unlike that *in vitro* work, it is impossible to isolate the septum from the hippocampus *in vivo*. Our strategy is to use the same chemical stimulus (KCl) to modulate a different, more distant afferent connection to the hippocampus. To choose a suitable target, we considered brain regions that are far away from the hippocampus and provide excitatory synaptic drive. Our primary candidate was the *nucleus reuniens* of the midline thalamus. The *nucleus reuniens* and the supramammillary nucleus are the two major external inputs from the diencephalon to the hippocampus^25^. These and other midline thalamic nuclei have been implicated in a number of important functions associated with the limbic system, including learning and memory^26–28^, but also have been shown to have direct modulatory effects on seizure activity in the limbic system^29,30^. However, the exact role of these connections is not known. We chose the *reuniens* for three main reasons: (1) its bilateral excitatory synaptic connections to CA1 of the dorsal and ventral hippocampus^25^, (2) published procedures for microinjection that produce synaptic activity in the hippocampus^31^, and (3) its deep midline location is far enough from the hippocampi to minimize potential diffusion of the microinjection into the hippocampus. We test the success of this method and by quantifying how much this method increases the risk of seizures above baseline levels and by rigorously comparing the induced seizures with spontaneous ones. Our results demonstrate that our method is capable of significantly increasing the risk of seizures during the injection times, and that the induced seizures are indistinguishable from spontaneous ones. This method provides a reliable *in vivo* method to modulate seizure threshold, allowing experimental control of timing of seizure onset and directed research into the mechanisms and effects of ictogenesis.

## Methods

### Animals

Adult male Sprague-Dawley rats were purchased from Charles River and kept individually housed under constant environmentally controlled conditions (12/12 h light-dark cycle, 22 °C) with access to food and water *ad libitum*. All animal procedures were performed in accordance with the University of Michigan animal care committee’s regulations, and we confirm that the entire procedure was approved by that committee.

### Pilocarpine-induced *status epilepticus*

Epileptic animals (n = 16) and sham controls (n = 12) were generated using a partially modified intraperitoneal pilocarpine model, similar to previous studies^32,33^. Briefly, 56-days-old rats were pretreated with atropine methylbromide (5 mg/kg ip; Sigma-Aldrich, St. Louis, MO) 20 min prior to pilocarpine hydrochloride (340 mg/kg IP; Sigma-Aldrich) for epileptic animals, or an equivalent volume of 0.9% saline for age-matched sham control animals. If seizure activity was not observed within 40 min after the initial pilocarpine dose, an additional dose of 170 mg/kg was given. Seizures were behaviorally monitored and after 90 min of *status epilepticus*, seizures were terminated with diazepam (10 mg/kg ip; Hospira, Lake Forest, IL). Sham controls were treated with diazepam 2 h after the saline injection. Spontaneous seizures were monitored for 14 days using a video-EEG recording system.

### Surgery

**Figure 1:**
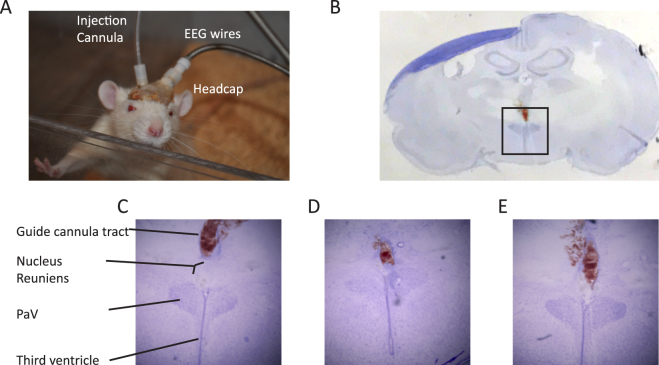
Verification of deep electrode and injection sites. (**A)** Photograph showing location of guide cannula (here with injection cannula inserted) and EEG headcap. (**B)** Low magnification Nissl stain with fluorescent DiI, showing relative location of the *reuniens*. The defining landmark is the dark, winged shape of the paraventricular hypothalamic nucleus (PaV), sitting astride the third ventricle. The *nucleus reuniens* is a loosely grouped nucleus just above the ventricle. Box: area shown in (**E**). Red stain is fluorescent DiI showing location of cannula. (**C–E)** Three rats show successful targeting of the *reuniens*, here marked by DiI fluorescent staining ending just atop the *reuniens* nucleus. All rats included in this study also had the placement of hippocampal depth electrodes verified in similar manner with DiI.

Epileptic and control rats were anesthetized with a ketamine/xylazine mixture (70 mg/kg ip and 10 mg/kg ip, respectively) and placed in a stereotactic frame (David Kopf Instrument, Tujunga, CA) two weeks after the pilocarpine injection. Using sterile surgical techniques, an incision was made midline on the scalp and the skull exposed. Four holes were made into the skull using a high-speed drill. Electrodes were positioned and fastened (left and right frontal, one cerebellar, and one reference over the sinus cavity) using mounting screws (E363/20; PlasticsOne, Roanoke, VA). Then, for the acquisition of local field potentials, two single channel depth electrodes (PlasticsOne, Roanoke, VA) were stereotactically implanted, one in the left hippocampus (AP −4.1, ML 2.3, DV −2.23) and one in the right hippocampus (AP −4.1, ML −2.3, DV −2.23). In two epileptic animals, a 26-gauge guide cannula (C315GA/SPC; PlasticsOne) was coupled to the depth electrode (PlasticsOne, Roanoke, VA) and stereotactically implanted in the left hippocampus (AP −4.1, ML 2.3, DV −2.23) to guide a 33-gauge injecting cannula (C315IA/SPC; PlasticsOne). In all cases, the sockets were fitted into a 6-pin electrode pedestal (MS363; PlasticsOne) and the entire apparatus was secured with dental cement. For local drug injection into the *nucleus reuniens*, we followed a previously described procedure that demonstrated that focal injection into the *reuniens* generated synaptic excitation in the hippocampi^31^. In short, the tip of a 26-gauge guiding cannula (C315GA/SPC; PlasticsOne) was painted with DiI (Molecular Probes by Life Technologies, Eugene, OR) and stereotactically implanted (AP −1.8, ML −1, DV −7.2, with a 15° arm angle from vertical axis) to guide a 33-gauge injecting cannula (C315IA/SPC; PlasticsOne). Once in place, the assembly was cemented to the skull (Fig. 1A). All animals received buprenorphine hydrochloride (0.05 mg/kg; Reckitt Benckiser Pharmaceuticals Inc., Richmond, VA) subcutaneously every 12 h for two days.

Only rats with confirmed placement in the hippocampus and the *nucleus reuniens* (Fig. 1B–E) were considered in the final analysis.

### Video/EEG monitoring and infusion procedures

During the two weeks of recovery from stereotactic surgery, no video/EEG monitoring was done. On day 13 post-surgery, epileptic and control animals were monitored for up to 48 h by continuous video/EEG recording (Ceegraph Vision; Bio-logic System Corporation). Fifteen days after the surgery, the experiment starts with “day 1” (see Table 1). Then, KCl (120 mM; Sigma-Aldrich, St. Louis, MO) was injected (0.1 µl/min over 5 min) into the *nucleus reuniens* of freely moving rats via the injecting cannula connected with polyethylene tubing to a micro syringe (Hamilton Co., Reno, NV). This injection was repeated 8 more times, with 15 minutes between injections, comprising 180 minutes (i.e. 9 periods of 5 + 15 minutes). Once the ninth administration was completed, the injection cannula was removed from the guide cannula but the animal remained connected to the video/EEG recording. The rationale for making 9 separate injections into *nucleus reuniens* over a 3-hour period was based on (1) the need to inject sufficient KCl to produce the effect, while minimizing the rate of administration to avoid volume expansion and (2) allowing time for the animal to recover between seizures, and (3) to eliminate the volume from the previous injection. In five animals, control injections with phosphate buffered saline (PBS) 1X solution (Fisher Scientific, Fair Lawn, NJ) were performed on a separate day using a new injection cannula under the same experimental protocol, randomly done either before or after the KCl experiments. Due to various technical and scheduling concerns, not all animals had identical experimental schedules. It is important to highlight that all experiments began at 10 AM and used the same protocol, and that there was always only a single experiment per 24-hour period (see Table 1). Recordings were sampled at 256 Hz and concurrent time-synched video was analyzed offline. Seizures were identified manually by an observer blinded to the groups assisted by EEG viewing software (Insight 12, Persyst Corp., San Diego, CA).

**Table 1:** Experimental design.

Rat #	Day of experiment (d)/Injection	# seizures 3 h before injection	# seizures during the 3-h experimental time	Change from baseline
**Epileptic 1**	d1/KCl	3	6	3
	d12/KCl	2	2	0
	d21/KCl	1	1	0
**Epileptic 2**	d1/KCl	0	0	0
**Epileptic 3**	d1/KCl	0	0	0
	d12/KCl	0	0	0
	d30/KCl	1	4	3
**Epileptic 4**	d1/KCl	1	4	3
	d20/KCl	1	7	6
	d31/KCl	1	0	0
**Epileptic 5**	d1/KCl	1	2	1
	d22/KCl	1	2	1
	d30/KCl	1	2	1
**Epileptic 6**	d1/KCl	0	2	2
	d21/PBS	0	0	0
**Epileptic 7**	d1/PBS	0	0	0
	d21/KCl	0	0	0
**Epileptic 8**	d1/KCl	0	2	2
	d5/PBS	0	0	0
**Epileptic 9**	d1/KCl	4	3	-1
	d2/PBS	3	3	0
**Epileptic 10**	d1/KCl	0	1	1
	d2/PBS	0	0	0
**Epileptic 11**	d1/KCl	0	2	2
**Epileptic 12**	d1/KCl	0	1	1
**Epileptic 13**	d1/KCl	0	0	0
**Epileptic 14**	d1/KCl	1	3	2
**Epileptic 15**	d1/KCl	1	3	2
	d4/electrical stimulation	0	0	0
	d12/Kainic acid	0	4	4
**Epileptic 16**	d1/KCl	1	1	0
	d7/electrical stimulation	0	4	4
	d15/Kainic acid	0	4	4

Detailed schedule of KCl or PBS injections into each epileptic animal. ‘D1’ is 15 days after implantation. All the animals were monitored for up to 48 h by continuous video/EEG recording before the experimental day, starting 13 days after the surgery. Once the ninth administration was completed and the injection cannula was removed from the guide cannula, the animals remained connected to the video/EEG recording one more day. All the experiments were performed under the same experimental protocol design. 13/16 epileptic animals had seizures during the KCl (120 mM) microinjections into *nucleus reuniens.* In total, seizures were observed in 18/24 individual KCl injections. The last column shows the individual changes (# of seizures) during the 3-h experimental time. No seizures were induced in the twelve control animals evaluated (data not shown).

#### Preliminary testing

The KCl concentration was selected based upon preliminary acute dose-response experiments in 20 rats that were not included in this analysis. In these experiments, 0.2–1 µL of KCl was injected over 1 minute at varying concentrations (20, 40, 80, 120, 160 mM). We determined that 120 mM was the minimum concentration that induced seizures in at least 25% of the experiments (data not shown). In addition, two epileptic animals were implanted with a special *reuniens* guide cannula that contained a pair of single channel depth electrodes (C315G-MS303/2/SPC; PlasticsOne) in order to record from within the *reuniens*. Both of these animals had seizures successfully induced by *reuniens* injection. Comparison of the *reuniens* recording with the hippocampal recordings revealed that the seizures did not arise within nor spread to the *reuniens*: the only ictal activity seen was far field propagation from the hippocampus (data not shown).

#### Alternative protocols

Several days after the KCl experiments, two animals were monitored again for up to 1 h with continuous video EEG, during which time we induced seizures by applying an electrical stimulus directly to the hippocampus, using a bipolar stimulus between the recording electrode and the frontal reference electrode (40 Hz square wave spike trains at 75 µA, 10 s duration, 2 ms pulse width, similar to our previous work^24^). One week later, the same animals were monitored again for up to 1 h by continuous video/EEG recording in order to assess the response to a direct chemoconvulsant application. Using the same equipment as before, kainic acid (0.4 µg/0.2 µl saline; Sigma-Aldrich, St. Louis, MO) was injected (0.1 µl/min over 1 min) into the left hippocampus of freely moving rats via the injecting cannula connected with polyethylene tubing to a micro syringe (Hamilton Co., Reno, NV). Note that these injection cannulas were inserted directly into the hippocampus via a separate guide cannula implanted into the hippocampus (i.e. independent of the guide cannula placed into the *reuniens*), as described above. This injection was done only once in order to induce seizure(s) via a direct chemoconvulsant. The goal of these two experiments was to compare these other methods of seizure induction (chemoconvulsant or electrical stimulation to the hippocampus) with the KCl/*reuniens* ictogenesis model described herein.

### Histology

At the completion of the experiments, rats were deeply anesthetized using isoflurane (Vet One, Boise, ID) inhalation and overdosed using an intraperitoneal injection of pentobarbital (Vortech Pharmaceuticals, Dearborn, MI). Following the loss of the righting reflex and lack of reflex withdrawal responses to toe-pad pressure, animals were transcardially perfused with 0.9% saline solution followed by a 4% paraformaldehyde (Sigma-Aldrich, St. Louis, MO) solution. Brains were then extracted and stored at 4 °C in a 4% paraformaldehyde solution for a minimum of 24 h. Then, the brains were washed four times with PBS 1X solution and stored at 4 °C in a 30% sucrose solution for 5 days. Brains were frozen with dry ice and sliced into 40-µm-thick coronal slices using a cryostat. The coronal sections were stained with cresyl violet for histological examination. Each electrode and cannula tip placement was verified to be in hippocampus or *nucleus reuniens*, respectively. The animals with unsuccessful placement were excluded from the study.

### Statistical analysis

#### Quantification of seizure risk: mean seizure rate

We determined the mean seizure rates for the three hours before (basal) and during the experiment. Seizure rates were defined as the mean number of seizures per hour, averaged over multiple experiments and animals. Assuming seizure initiation is a Poisson process^34^, the variance on the number of seizures is equal to the number of seizures. The significance of the change between basal and experimental seizure rates can thus be computed using a χ^2^ statistic with one degree of freedom. Rarely, some animals had a very high basal seizure rate (≥1 seizure/hour) prior to the experiment. With such a high rate, these animals were likely already at or near the seizure threshold (no animal ever had a rate over 2/hour), making it difficult to show an increase in seizure rate. Nevertheless, to be robust these animals were included in the analysis. A separate analysis redacting these experiments (Epileptic 9 and Epileptic 1 day 1) had similar results (data not shown).

#### Quantification of seizure risk: time-dependent hazard rate

With dynamic processes such as seizures, another useful measurement is to determine how risk changes with time. In order to investigate the time-dependence, a kernel-smoothed Nelson-Aalan estimate of the seizure hazard as a function of time was computed from three hours before until five hours after the start of each experiment, as performed previously to assess seizure risk^35,36^. This estimate requires definition of the number of experiments in which an animal is at risk of seizing as a function of time; we defined this as the number of experiments in which there was not a seizure in the 5 minutes preceding each given point in time. The Epanechnikov kernel was used for smoothing, with a bandwidth of 5/(sqrt (*N*)) hours, with *N* being the total number of seizures used to compute the hazard function. The hazard estimator uncertainty was computed using standard statistical methods^35^. We additionally computed the time where the experimental seizure hazard rate was most significantly different than the baseline rate, again using a χ^2^ statistic.

#### Seizure selection

For the analysis below, the first two hippocampal seizures induced by KCl injection into the *reuniens*, and up to ten consecutive spontaneous seizures that were at least 3 hours before or 6 hours after the experiment were selected. All seizures were recorded on the same hippocampal depth electrode. No other selection criteria were used (e.g. seizures with different semiology or characteristics were all included). Five of the animals had fewer than 10 spontaneous seizures recorded (Epileptic 3, 6, 11, 14, and 16 had 9, 6, 6, 6 and 7 seizures respectively), so in those cases all spontaneous seizures were used. Epileptic 12 and 16 only had one seizure induced by KCl.

#### Qualitative visual seizure comparison

It is critical in a true model of ictogenesis that the induced seizures be analogous to the spontaneous ones. However, determining the similarity between two seizures is a difficult task. We first used standard clinical interpretation, which is based upon qualitative visual features of the video and EEG. We measured the clinical semiology of each seizure using the Racine scale^37^ by observing the video-EEG recording. A clinical epileptologist then compared the location, timing, and visual features of each induced seizure with several spontaneous seizures from each animal individually. EEG appearance was also compared using spectrograms: one channel (hippocampal depth) was used to create a Morlet plot demonstrating the spectral power versus time during each seizure, utilizing a scaled ‘continuous wavelet transform using FFT’ (cwtft) function in Matlab (Mathworks). Each Morlet plot was visually inspected and compared. Any clear differences between the induced and spontaneous seizures were noted; seizures that appeared to be different morphologically were considered to be distinct from the spontaneous events. The plots are shown from the beginning (t = 0) to the end of each seizure, which allows analysis of the entire seizure.

#### Quantitative seizure comparison

In addition to those visual measurements, we compared the seizure characteristics using a battery of quantitative tools. The goal was to classify whether any given seizure was similar to a specific set of spontaneous seizures.

We calculated the following features on each seizure independently, with separate measurements for the entire seizure, first 10 seconds, and last 10 seconds: delta (0.5–4 Hz), alpha (8.5–13 Hz), beta (13–30 Hz) and gamma (30–128 Hz) band power; alpha-delta ratio; peak frequency; line length; number, median interval, median amplitude of spikes (positive and negative); mean and standard deviation of Teager-Kaiser Energy; and seizure length. Delta power was only evaluated in the first 10 seconds as it is dominated by movement artifact afterwards, and seizure length only for the entire seizure. This created a vector of 44 features that described each seizure. We then reduced these features in each animal individually using a standard principal components analysis (PCA, (Matlab ‘pca’ function), after first normalizing the features using the mean and standard deviation of the features from all of that animal’s seizures. We then used a one-class support vector machine (SVM) to characterize the distribution of *only the spontaneous seizures* of each animal (Matlab ‘fitcsvm’ function, with outlier fraction of 0.05, radial basis kernel with scale = 50), using the first two principle components. We then classified each individual seizure whether it was a member of this “spontaneous class” using the normalization and PCA coefficients and trained SVM (Matlab ‘predict’ function) of that given animal.

In summary, this process requires four steps: calculate features for each seizure, reduce the dimensionality of the features, train an algorithm (one-class SVM) to identify spontaneous-like seizures of a given animal, and then classify all seizures regarding whether they are similar to those spontaneous seizures. This method allowed us to compare the KCl-*reuniens* seizures with the spontaneous seizures, as well as seizures from different animals with each other. We tabulated how many seizures of each type were classified as similar to the spontaneous distribution, and calculated the uncertainty based upon Poisson statistics (uncertainty = sqrt(number of seizures classified as similar)/(total number)). Significance was calculated using χ^2^ statistic.

## Results

### KCl microinjection to the *nucleus reuniens* induces hippocampal seizures

Table 1 shows in detail the schedule of KCl or PBS injections into each epileptic animal. On the first attempt of focal microinjections of KCl (120 mM) in the epileptic group, 75% of the subjects (12/16) had seizures (Table 1). Including injections on subsequent days, 13/16 animals (81%) had seizures at least in one trial. In total, seizures were observed in 18/24 individual KCl-*reuniens* injections (75%).

No control animals had seizures during the injections or at any other time (data not shown). Seizures only occurred in one epileptic animal during PBS injection, though this animal had the same rate of seizures (1/hour) even before the injection (Epileptic 9). The other epileptic animals with PBS injections (Epileptic 6, 7, 8, and 10) did indeed have spontaneous seizures, but there were none in the 3 hours prior or during that injection. Note that all animals were recorded for 24–48 hours prior to the injections. As is expected for the pilocarpine model, the seizure rates on different days were quite variable in many animals, which did not allow identification of a stable baseline over long periods. The three hours prior to seizure onset were chosen as the baseline in order to assure comparison with similar brain states.

### Quantifying similarity between induced and spontaneous seizures

**Figure 2:**
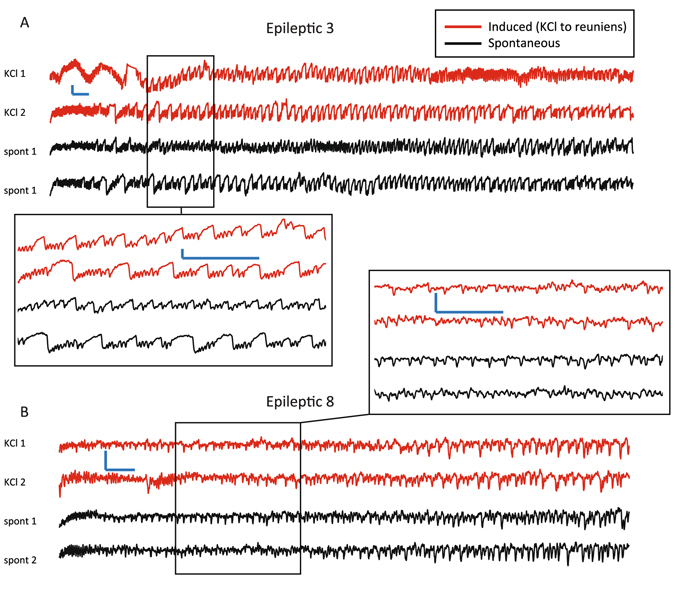
Seizures occurring during the KCl injections versus spontaneous seizures, raw data. Four separate seizures are shown for Epileptic 3 (**A** and left inset) and Epileptic 8 (**B** and right inset). Note each tracing is from the same hippocampal depth electrode during a different seizure. In each animal, both seizures occurring during the KCl microinjection (KCl 1, 2) into the *nucleus reuniens* (top, red) have very similar morphology to the spontaneous seizures (spont 1, 2) recorded on other days in the same animal (black). Note that in both animals there were no spontaneous seizures in the 3 hours prior to the experiment; these spontaneous seizures occurred in the 1–2 days previous. Scale bars: 500 μV, 1 s. The time scale was chosen to optimize the ability to evaluate the dynamical behavior of the individual spikes within the seizure. Note that seizures in each rat have distinct features such as initial deflection, amplitude, frequency, etc, which are preserved in the seizures occurring during the KCl injections. Supplementary material shows more data and detailed explanations for all animals, including these.

**Figure 3:**
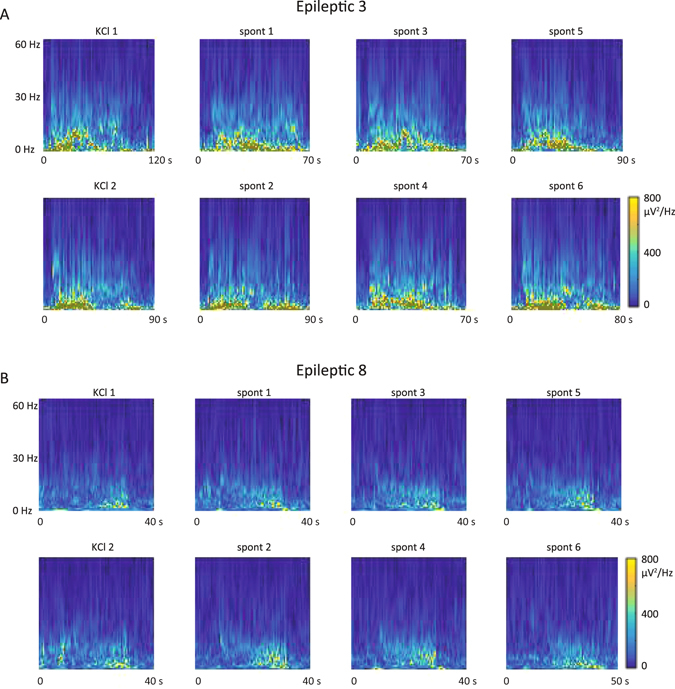
Seizures occurring during the KCl injections versus spontaneous seizures, frequency data. Seizures from Epileptic 3 (**A**) and Epileptic 8 (**B**) were converted into Morlet plots, which show the change in spectral power over time. The plots show data from each seizure. In each animal, there are two seizures occurring during the KCl injections and six spontaneous seizures shown; the first two columns correspond to the raw data in Fig. 2. Each rat has a distinct spectral pattern during their seizures, with some variability between different events. It is clear that the seizures occurring during the KCl injections in each rat are similar to their own spontaneous seizures, but different from the other animal’s seizures. X-axes show duration of each seizure, also including 10 s of EEG before and after the seizure. All plots have the same y-axis (0 to 60 Hz) and color scale. Additional data from these and all other Epileptic animals are included in the Supplement.

The preceding results suggest that this method increases the likelihood of a seizure occurring during the injections. It is then crucial to verify whether the seizures occurring during the injections are analogous to spontaneous ones. Doing so will demonstrate that this model recapitulates spontaneous seizures, which is distinct from the currently-existing methods of seizure induction (chemoconvulsants, kindling). We first verified in all animals that the seizures occurring during the injections had similar EEG appearance (Fig. 2). Additional detailed analysis on seizure comparisons from all animals are included in the supplementary material. All seizures identified were validated to have clear behavioral and electrographic evidence of seizures. We validated that the induced seizures produced ictal behaviors that were identical to those of the spontaneous seizures based upon the Racine scale (e.g. behavioral arrest, rearing, falling)^37^. After viewing the raw EEG and video of all seizures, there was no appreciable difference in the EEG appearance or semiology between the seizures occurring during the injections and spontaneous seizures, beyond the typical variability seen within spontaneous seizures (e.g. different types of seizures seen in the same animal, such as occasional behavioral arrest interspersed with rearing and convulsions). For a more objective visual comparison, we also conducted a time-frequency analysis using Morlet plots and compared the seizures occurring during the injections and spontaneous seizures (Fig. 3 and Supplementary Material). These plots provide a visual demonstration of the frequency content over time; brighter colors indicate stronger power. Analysis of these plots typically focuses on comparing the visual differences and similarities that correspond to the distribution and temporal evolution of spectral power. In Fig. 3, Epileptic 3 begins the seizure with high spectral power up to 30 Hz, which diminishes during the seizure, whereas Epileptic 8 starts with very little spectral power but towards the end has high power up to 10 Hz. With these additional data, we found that only in three animals (Epileptic 5, 6, 16) was there any appreciable difference between the seizures occurring during the injections and spontaneous seizures, and the differences were subtle. We found that animals have some variability in their spontaneous seizures, and that the seizures occurring during the injections appear to be very similar to that group. Of note, some of the animals (e.g. Epileptic 4) had spontaneous seizures with a wide range of morphologies, some of which appeared to be outliers from the rest of the spontaneous group. In addition, it is clear that different animals have distinct types of seizures from each other: both the raw EEG and the Morlet plots show that the seizures from subject to subject are quite different across animals. These results were true across all Epileptic animals: the seizures occurring during the injections from a given subject were qualitatively indistinguishable from its own spontaneous ones, but different from other animals’ seizures. These results suggest that the events occurring during the injections are indeed recapitulating spontaneous seizures within a given animal.

**Figure 4:**
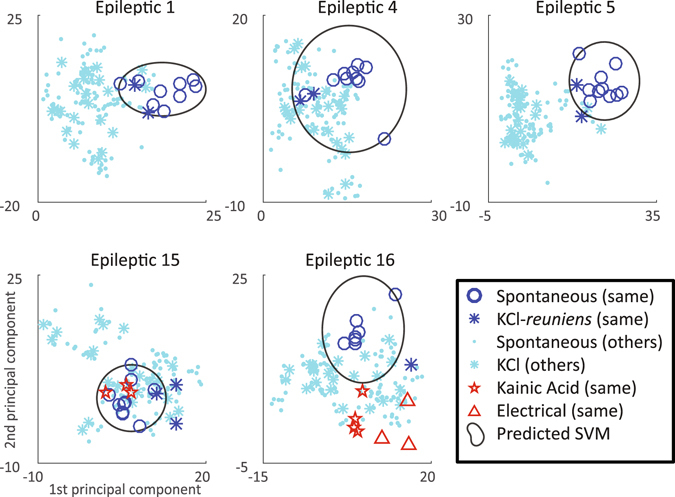
Comparison of feature distributions. A set of 44 features was calculated for each seizure, then reduced to two dimensions with principal components analysis. Plots show the location of each seizure’s first two components. The set of spontaneous seizures in a given animal was used to determine the one-class SVM (black line). Each seizure was then compared with this threshold to determine if it belonged within the spontaneous seizures’ classification. We compared all seizures from the same animal (dark blue) and other animals (light blue). Seizures occurring during the KCl-*reuniens* injections (dark blue *) were indistinguishable from spontaneous seizures of the same animal (Dark blue circle) in most animals (top row). A small number of seizures occurring during the KCl injections were distinct from their spontaneous groups (bottom row). Most kainic acid-induced seizures (red stars) and all electrical-induced seizures (red triangles) were distinct from spontaneous seizures, as were the majority of induced and spontaneous seizures from other animals (light blue * and circles).

For a more robust comparison, we evaluated the quantitative characteristics of the EEG recordings. This type of strategy was recently used to compare induced versus spontaneous generalized seizures using microelecrodes^38^. We calculated a battery of features for each seizure, then trained a machine learning algorithm (one-class SVM), using spontaneous seizures of a given animal, to classify whether each seizure (from all animals) was similar to those spontaneous seizures (Fig. 4). The range of cases expected in a set of spontaneous seizures is observable in this figure: in some animals the seizures were very stereotyped and the features tightly clustered, quite distinct from the seizures in other animals (e.g. Epileptic 1 and 5). In contrast, some animals have a broader distribution that encompasses seizures from many other animals (Epileptic 15), though sometimes the broad classification was due to single spontaneous seizures that were very different from the rest (Epileptic 4, 16).

After delineating the range of spontaneous events with the one-class SVM, we can compare all other seizures by determining whether they lie within the SVM boundary for each individual animal. We compared all combinations of seizures across all animals: in other words, we plotted all the seizures occurring during the injections and the spontaneous seizures from all 13 animals onto the distribution and SVM for each animal. As seen in Table 2, 68% of the seizures occurring during the KCl-*reuniens* injections were similar to each animal’s own spontaneous seizures (n = 25). While this value was not ideal, it is important to note that some spontaneous seizures are outliers themselves, as only 91% of them were successfully classified using the same analysis. In addition, we compared all permutations to determine how similar the animals were to each other: 25% of spontaneous (n = 1368) and 24% of the seizures occurring during the KCl-*reuniens* injections (n = 300) were similar to the spontaneous seizures in different animals. Using the 91% as the gold standard, the seizures occurring during the KCl injections were statistically indistinguishable from the spontaneous seizures in a given animal (χ^2^, p = 0.22), while across different animals both KCl and spontaneous seizures were different (p < 0.01). This latter comparison shows not only that the seizures occurring during the KCl injections are similar to a given animal’s own seizures, but also that different animals have different seizures and this method can identify those differences. As a negative control, we also compared the features of seizures induced by direct kainic acid injection and electrical stimulation to the hippocampus. Electrographically and semiologically, the seizures were obviously distinct from spontaneous seizures—kainic acid induced long, repeated seizures with different semiology and EEG morphology, and electrical stimulation induced low voltage fast activity without the typical spike waves, and subtle clinical findings. The feature analysis corroborated this: only 43% (p = 0.07) of kainic acid and 0% (p < 0.01) of electrical seizures were similar to the spontaneous ones (Table 2). In summary, in a given animal the KCl-*reuniens* method induced seizures that were similar to that animal’s own spontaneous seizures. Visual analysis of the EEG, video, and spectrograms suggested the seizures were identical, and a quantitative analysis found that most seizures induced by this method are statistically indistinguishable from spontaneous seizures. In addition, previous methods of inducing seizures (direct application of chemoconvulsants or electrical stimulation) are unsuccessful in producing similar seizures. In contrast, seizures in different animals are usually easy to distinguish from each other. Although in general most of the seizures were characterized by similar fast spiking activity, each animal tended to have seizures with a stereotypical, fairly unique appearance. These differences are typical for the variability between subjects, and could be the result of individual anatomy, specific pathology, electrode placement, etc. Based upon these qualitative and quantitative analyses, we conclude that the induced seizures are indeed representative of each animal’s typical seizures.

**Table 2:** Comparison of seizure features

	A: Own seizures		B: Other animals’ seizures	
	% similar (N)	p value	% similar (N)	p value
Spontaneous	0.91±0.09 (114)	1.00	0.25±0.01 (1368)	0.00
KCl-*reuniens*	0.68±0.17 (25)	0.22	0.24±0.03 (300)	0.00
Kainic acid (hippoc)	0.43 ± 0.25 (7)	0.07	0.21±0.05 (84)	0.00
Electrical (hippoc)	0.00 ± 0 (3)	0.00	0.14±0.106 (36)	0.00

Tabulated results for quantitative seizure comparison. After determining the distribution of each animal’s spontaneous seizures (see Fig. 4), every seizure from every animal was compared to that distribution. Column A: seizures occurring during the KCl-*reuniens* injection method were not significantly different from the spontaneous seizures, while those induced by both kainic acid and electrical stimulation were different (χ^2^). Column B: when comparing seizures across different animals, other animals’ seizures were very different each animal’s own spontaneous seizures. These results prove that seizures are different across different animals and that the seizures occurring during the KCl-*reuniens* method are similar to a given animal’s own seizures.

### *Nucleus reuniens* KCl injections increase hippocampal seizure risk

As seen in Table 1, the majority of epileptic animals had seizures during the KCl injections, but it is important to assess the baseline seizure rate to determine whether the injection is truly increasing the risk of seizures. We evaluated the efficacy of this ictogenesis model in two ways: comparing mean seizure rate and comparing seizure hazard rate.

Table 3 shows the mean estimated basal and experimental seizure rates and the significance of the difference between them (χ^2^), demonstrating that KCl injections significantly increased the mean seizure rate, while PBS did not. In the PBS injections, no seizures were induced in any of the four animals analyzed. Epileptic 9 did have three seizures during the PBS injections, but that was unchanged from its high baseline rate. The control (non-epileptic) animals never seized during the experiments nor during the basal periods.

**Table 3:** Seizure rate.

Group	Injection	Number of experiments	Baseline seizure rate	Experimental seizure rate	*p* value
Epileptic (n = 16)	KCl	24	0.28 ± 0.06	0.67 ± 0.10	0.0007
Epileptic	PBS	5	0.2 ± 0.12	0.2 ± 0.12	1

Mean seizure rate for each experiment (in seizures/hour), for 3-hour intervals during and before the experiment. The potassium chloride (KCl) injections induced an increased seizure rate, while there was no effect from PBS injections.

**Figure 5:**
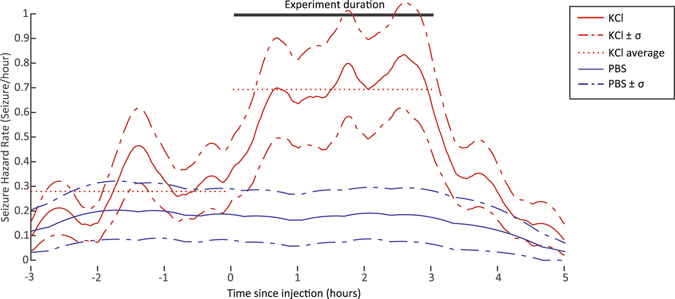
Time-dependent seizure hazard rates. The KCl solution injection significantly increases the seizure hazard rate immediately after the first injection was administered (time 0), more than doubling the risk of seizures during the experiment. In contrast, PBS injections had no effect on seizure risk. Dotted lines indicate the average risk across all animals before and during the injections.

Given the dynamic nature of seizures, we also evaluated the time-dependent hazard rates during the experiments (Fig. 5). During the KCl injection, there is a clear increase in the risk of seizures, starting immediately after the injections start and steadily increasing throughout the 3-hour experiment. This change is significantly different from the pre-experiment baseline (*p* < 0.01). These results suggest that there is an additive effect of the microinjections over time.

In summary, this ictogenesis method significantly increases the risk of seizures during a defined experimental period. The induced seizures are indistinguishable from the spontaneous ones. Thus this model successfully modulates seizure threshold and provides a controlled means of controlling ictogenesis.

## Discussion

Clinically, the primary goal of managing seizures is to lower the risk that they occur. However, in order to test antiseizure therapies and investigate biomarkers of epilepsy, it is prudent to have a method to *increase* the risk of seizures. It is well known that certain conditions increase seizure risk (e.g. sleep deprivation, medication withdrawal, etc), creating “pro-ictal” epochs during which seizures are more likely to occur, but still with paroxysmal onset times. These pro-ictal states, which may or may not result in a seizure, pose a significant challenge in the search for seizure biomarkers: how does one assess the biomarker with a “near miss” when a seizure does not occur? It is impossible to know whether a biomarker correctly identified a true pro-ictal state that resolved (true positive) or did not have a reliable correlation with seizure precursors (false positive). Thus, in order to model and investigate physiological ictogenesis, it is important to be able to modulate the risk of seizures in a controlled fashion. An expected outcome of such a method is that, depending on the strength of the stimulus and the state of the subject, it may not produce seizures every time. A method capable of modulating the risk of endogenous seizures would provide a tremendous opportunity to investigate the mechanisms and biomarkers of ictogenesis. This is in contrast to kindling and chemoconvulsants, which are applied at suprathreshold levels to induce seizures immediately. Those methods reliably induce seizures at precise times, but they produce non-physiological seizures, and they fail to recapitulate the natural progression of ictogenesis. The goal of our model was to increase the risk of seizures but not force them to occur, which means that seizures might not be produced every time. Our model excites a seizure focus (the hippocampus) via physiological pathways (afferent synaptic activity from the *reuniens*) and makes seizures more likely over a set period of time, simulating epochs of a pro-ictal state. The seizures occurring during the KCl injections are indistinguishable from a subject’s specific spontaneous seizures. Our data show that this new method increases seizure risk, providing controlled conditions in which to test many potential new avenues of research into ictogenesis and seizure control.

How a seizure begins has been a longstanding question for clinicians and researchers. Given current technological limitations and the vast spatiotemporal scale that seizures embody, it is impossible to characterize the mechanisms of ictogenesis fully —thus the pressing need for experimental models. The question of whether a given experimental mechanism “causes” ictogenesis remains profoundly difficult to prove—especially since we still have little understanding of naturally occurring seizures. Likewise, it is difficult to prove that any given seizure is “the same” as another—the best we can do is assess whether the seizure looks and acts the same. Most prior seizure research has relied upon qualitative comparisons for that task: visual interpretation of EEG and Racine scale. Unfortunately, those measurements are very coarse. For this reason, we and others^38^ have taken a quantitative approach to analyze EEG features, but these are still limited. One of the primary goals of this model is to provide a controlled environment in which to test more detailed questions of the mechanisms of ictogenesis, once higher resolution technology becomes available.

There are compelling reasons to believe synaptic noise is a major player in ictogenesis. Most seizures appear to arise at random without any clear instigation, with the uncommon exceptions such as photosensitive and reflex epilepsies. But those rare exceptions provide an important insight into ictogenesis: it is clear that afferent sensory input into the cortical neurons is capable of inducing seizures. It is not hard to conceive of a host of conditions in which an epileptic focus will receive paroxysms of increased afferent activity, whether it be activity from other brain regions, changes in synaptic plasticity, amplification via axonal sprouting, etc. At times that input may arrive as a synchronous, deterministic barrage, such as with photic stimulation. But given the high degree of convergence and divergence of synaptic connections in the brain, it is more likely that afferent input is disorganized and asynchronous. From the point of view of a single cortical neuron, the sum of all its inputs can be described as synaptic noise. It is likely not coincidental that conditions that increase synaptic noise, such as the barrages of synaptic activity comprising the “UP state” during non-REM sleep^39–41^, or disrupted inhibition due to sleep deprivation^42^, are also known to increase the risk of seizures. However, in these conditions synaptic inputs do not necessarily arrive asynchronously. Any spatial or temporal synchrony of the inputs (i.e. gap junctions, recurrent synapses, or divergent connections from individual axons) will make it even more likely that the receiving neurons will cross threshold when subjected to this more correlated input. This effect was shown previously in normal signal detection^22^, high frequency oscillations^17^, and seizures^24^.

We hypothesize that increased synaptic noise is one of the many potential mechanisms by which an epileptic focus is driven beyond the seizure threshold. This hypothesis is based upon a broad spectrum of computational and experimental data. Within dynamical systems, noise is an effective method to push a neural network into different states^43^. Noise also is known to improve detection of subthreshold signals via Stochastic Resonance^44^, and to induce emergent coherent oscillations via Coherence Resonance^45^. Experimentally, synaptic noise improves signal detection in many types of peripheral and central neurons^21,23,46–48^. Many computational simulations have shown that synaptic noise synchronizes coupled neurons to form emergent oscillations^12,13,15,17,22,45,49–51^. Those oscillations arise suddenly when noise pushes the system across a threshold, in a manner very similar to the transition from resting state into seizures. Using this same concept in the intact septo-hippocampal formation, recent work showed that increased synaptic noise was present prior to spontaneous seizures and also that it was sufficient to induce seizures when experimentally modulated^24^. However, that work also demonstrated the seizures could be triggered *without* synaptic noise. Thus, it is likely that synaptic noise is just one of many potential triggers of ictogenesis. However, it is very difficult to evaluate the role of noise in *in vivo* ictogenesis due to technological limitations as well as the previous lack of an appropriate model. Our model provides a means for such experiments, but will require prolonged *in vivo* whole cell recordings of pyramidal cells, as synaptic noise is not reliably recorded by local field potentials or cell-attached recordings, even in brain slices^24^. When such recordings are available, future work could utilize this technique to monitor synaptic connectivity between the *reuniens* and hippocampus. It could also be adapted to many different brain regions: different levels of KCl will vary the synaptic drive from any afferent connection, providing a means to test the magnitude and role of synaptic connectivity. Thus, this method can be used to explore the physiological range of synaptic connectivity in addition to the thresholds needed for phenomena such as Stochastic Resonance, Coherence Resonance, and ictogenesis.

A viable model of *in vivo* ictogenesis must allow experimental modulation of the timing of seizures, and the seizures should be identical to the spontaneous ones. We believe the most appropriate method of doing so is to control an endogenous process that is likely to trigger seizures. Our paradigm to trigger ictogenesis is to control synaptic activity by modulating a region of brain upstream of the seizure focus. One method to do this is to force all upstream neurons to fire synchronously, such as with electrical stimulation or optogenetic triggering. However, this instantaneous barrage is unlikely to be the most common physiological mechanism for spontaneous seizures. A more general and physiological case is for synaptic noise to increase, such as when an upstream region becomes more active. Producing this effect experimentally is challenging. One technique is to apply a DC current to the upstream region, slightly depolarizing the cells and increasing their propensity to fire randomly, as it has been shown previously in hippocampal slices^46^, but this is difficult to perform *in vivo* due to tissue damage and electrode properties. Another method is focal drug injection to an upstream region, but must assure that the drug does not diffuse into the seizure focus itself. The *in vitro* preparation in Jirsa *et al*. (2014) used a specialized chamber to isolate the septum from the hippocampus. As isolating the septum is not possible *in vivo*, we targeted the *nucleus reuniens*, a midline structure that sends extensive excitatory synaptic contacts to the hippocampi^25^. It is ideal for this experiment because it is distant from the seizure focus, lying anterior, inferior, and medial to the hippocampi^52^, which minimizes the risk of diffusion from the injection site.

This experiment utilized the pilocarpine model, while Jirsa *et al*. (2014) *in vitro* work used low magnesium. However, our ictogenesis method is not specific to any particular model. In the hippocampus, we expect other upstream regions such as the septum and endopiriform nucleus would have similar results. Similarly, other focal seizure models could be adapted with this same strategy. We expect that any strategy to modulate upstream synaptic noise has the capacity of increasing the risk of focal seizures, allowing experimental control of ictogenesis. There were some challenges and drawbacks to this model. Placing the guide cannula is difficult and there is risk of bleeding, though it follows typical stereotactic protocols. Inserting the injection cannula and performing the experiment requires constant supervision, and the limits on the injected volume also limit the efficacy of the KCl. Future work will explore other methods of inducing noise, such as sub-maximal optogenetic triggering or DC current, that might require less technical supervision. This work serves as a proof of principle that seizures can be modulated in this fashion, opening the door for many other potential ictogenesis models.

In conclusion, it is critical to develop reliable models of ictogenesis, but to date they have been elusive. This work demonstrates a novel model that is capable of increasing the risk of seizures in temporal lobe epilepsy, providing experimental control of seizure threshold via endogenous pathways that mirror normal brain activity. With this method we can explore the basic mechanisms of ictogenesis, search for biomarkers associated with that risk in a controlled fashion, and potentially develop and optimize more effective antiseizure therapies.

## Electronic supplementary material

Supplementary material
