# Author Correction: Control of *in vivo* ictogenesis via endogenous synaptic pathways

**DOI:** 10.1038/s41598-017-17038-1

**Published:** 2017-12-06

**Authors:** Hiram Luna-Munguia, Phillip Starski, Wu Chen, Stephen Gliske, William C. Stacey

**Affiliations:** 1 Department of Neurology, University of Michigan, Ann Arbor, MI 48109 USA; 2 Department of Biomedical Engineering, University of Michigan, Ann Arbor, MI 48109 USA

*Scientific Reports* **7**:1311; doi:10.1038/s41598-017-01450-8; Article published online 02 May 2017

The original version of this Article contained a typographical error in the spelling of the author Phillip Starski which was incorrectly given as Philip Starski. This has now been corrected in the PDF and HTML versions of the Article.

