# Supplementary material for: Control of in vivo ictogenesis via endogenous synaptic pathways
Source: Sci Rep. 2017 May 2;7:1311. doi: 10.1038/s41598-017-01450-8 (PMC5431002; doi:10.1038/s41598-017-01450-8)
Supplement: Supplementary file 1 — Supplementary material [file 41598_2017_1450_MOESM1_ESM.pdf]

Control of *in vivo* ictogenesis via endogenous synaptic pathways

Hiram Luna-Munguia[1], Philip Starski[1], Wu Chen[1], Stephen Gliske[1], and William C. Stacey[1,2,*]

[1]Department of Neurology, University of Michigan, Ann Arbor, MI, 48109, U.S.A.

[2]Department of Biomedical Engineering, University of Michigan, Ann Arbor, MI, 48109, U.S.A.

*Corresponding author. william.stacey@umich.edu

Supplemental material

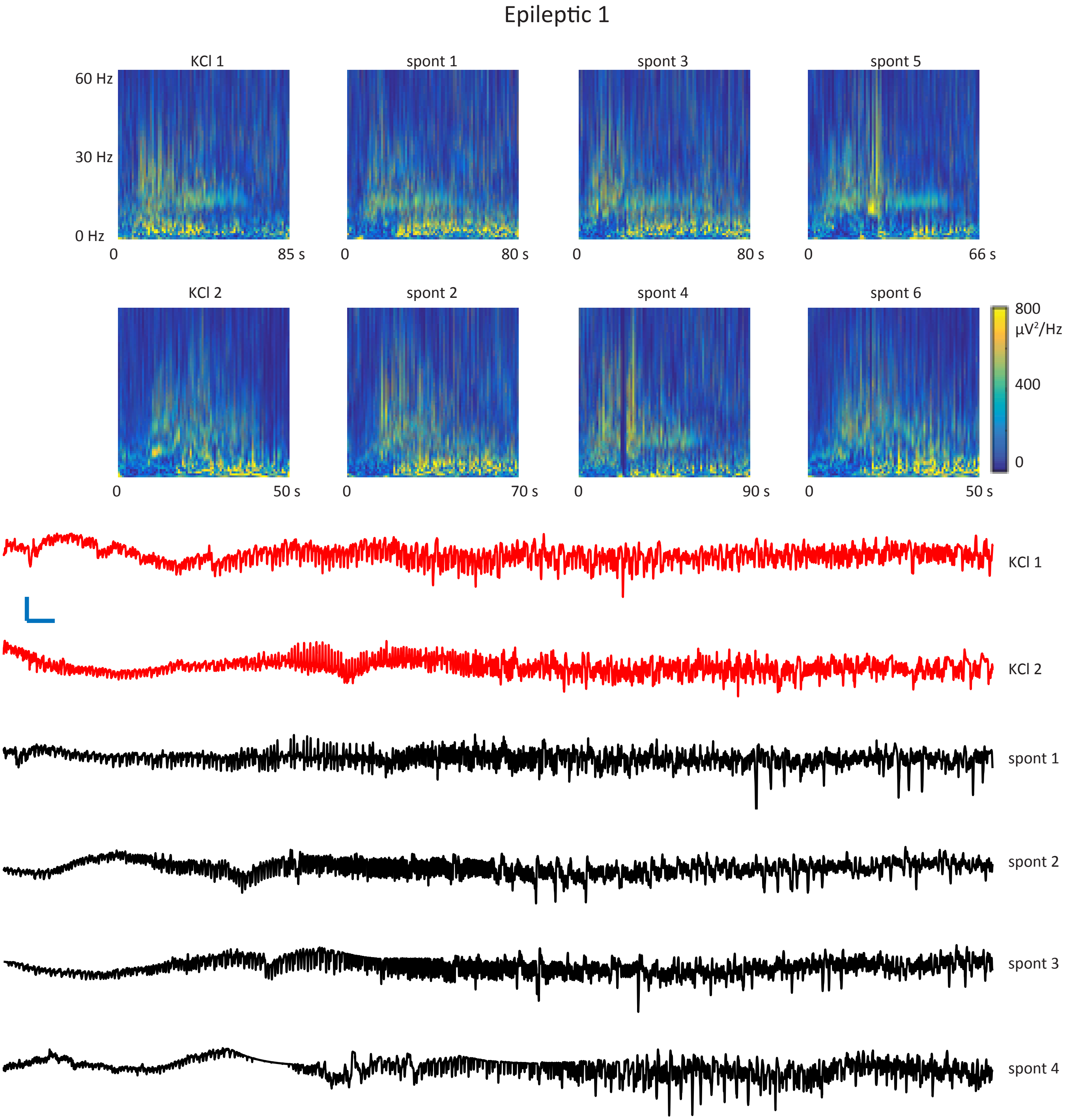

Morlet plots (top) and raw data (bottom) in Epileptic 1. KCl-*reuniens* induced seizures (first column on top, red tracings on bottom) are very similar to the spontaneous seizures. All seizures start with low voltage fast activity that evolves. The spontaneous seizures appear indistinguishable from several of the spontaneous ones, while there is some variability in the spontaneous .
Scale bars: 0.5 mV, 1 s

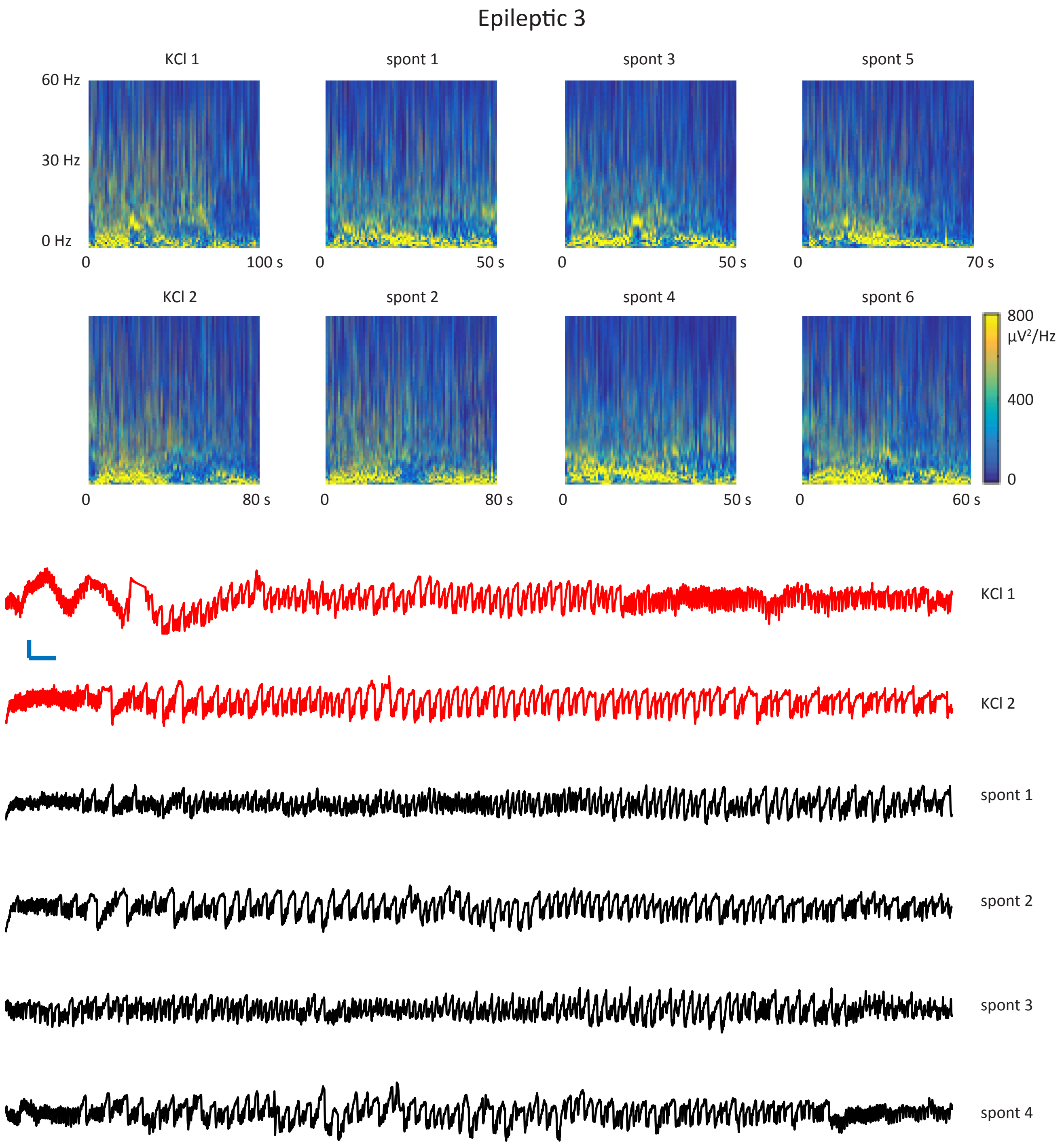

Morlet plots (top) and raw data (bottom) in Epileptic 3. KCl-*reuniens* induced seizures have the same repetitive theta firing, duration, and morphology as the spontaneous seizures. Scale bars: 0.5 mV, 1 s

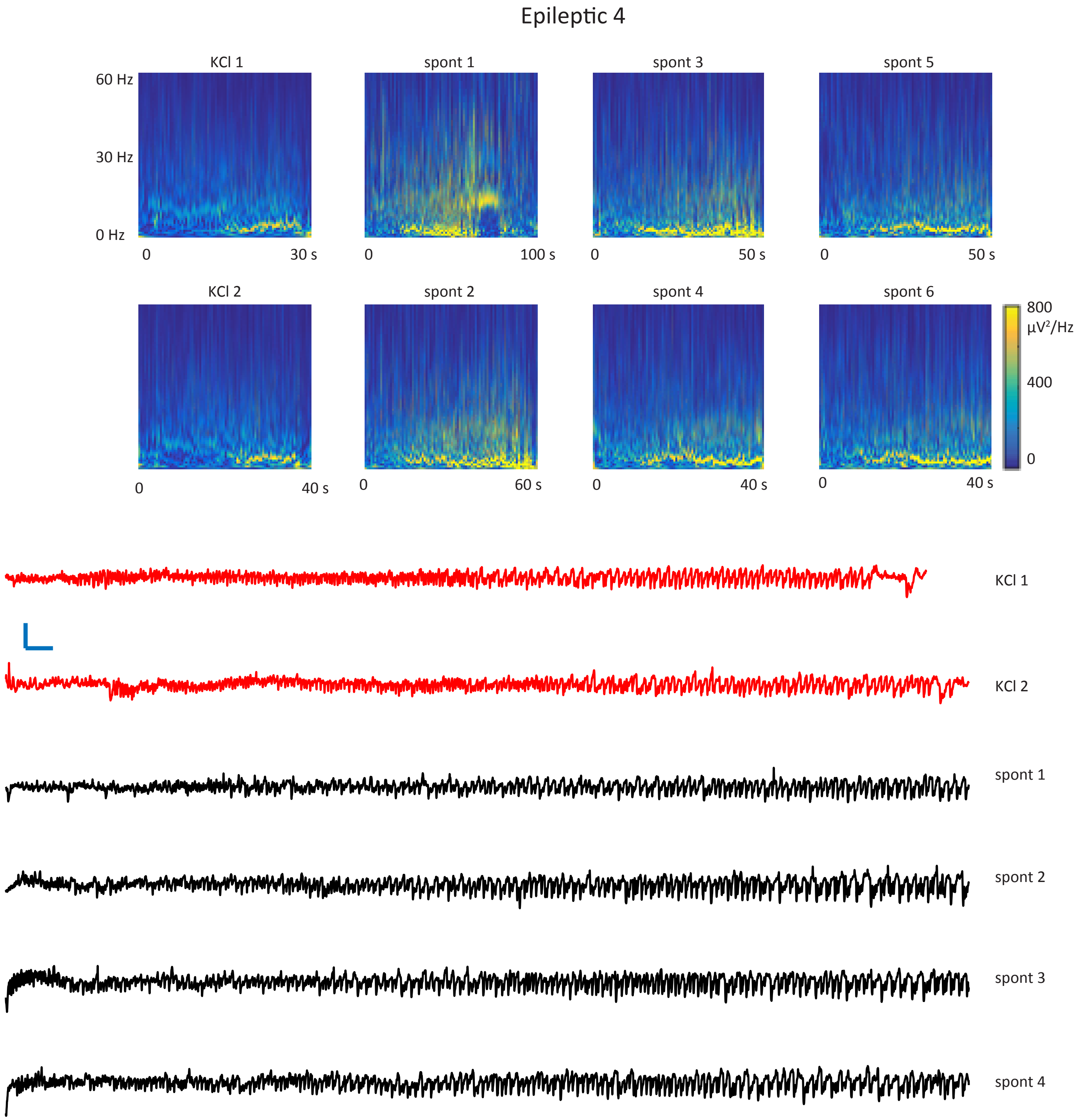

Morlet plots (top) and raw data (bottom) in Epileptic 4. 'Spont 1' is much longer than the rest and has some different dynamics at the end (fast spiking at 80 s, not shown), but the early fast spiking morphology is common among all seizures at the beginning. 'Spont 1' is actually quite different from the rest of the spontaneous seizures in the quantitative analysis:
in this case the KCl-induced seizures were more similar to the other spontaneous seizures than Spont 1 was. Scale bars: 0.5 mV, 1 s

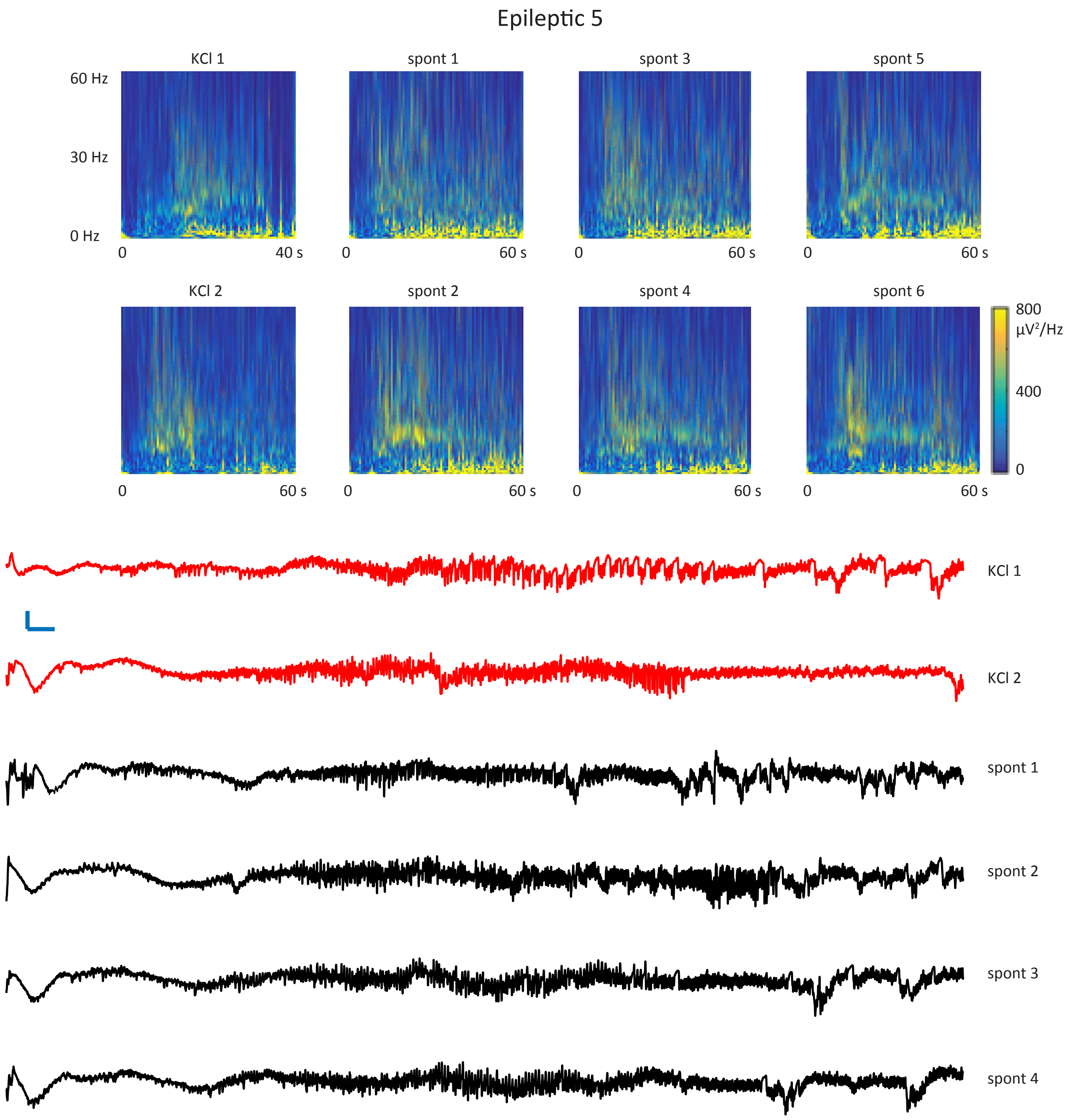

Morlet plots (top) and raw data (bottom) in Epileptic 5. Both spontaneous seizures initiate with the same delta waves and fast spiking as the spontaneous seizures. After 30 sec, there are some differences: 'KCl 1' has some repetitive firing and 'KCl 2' becomes flat; however, the quantitative analysis deemed that both were similar to the spontaneous group. Scale bars: 0.5 mV, 1 s

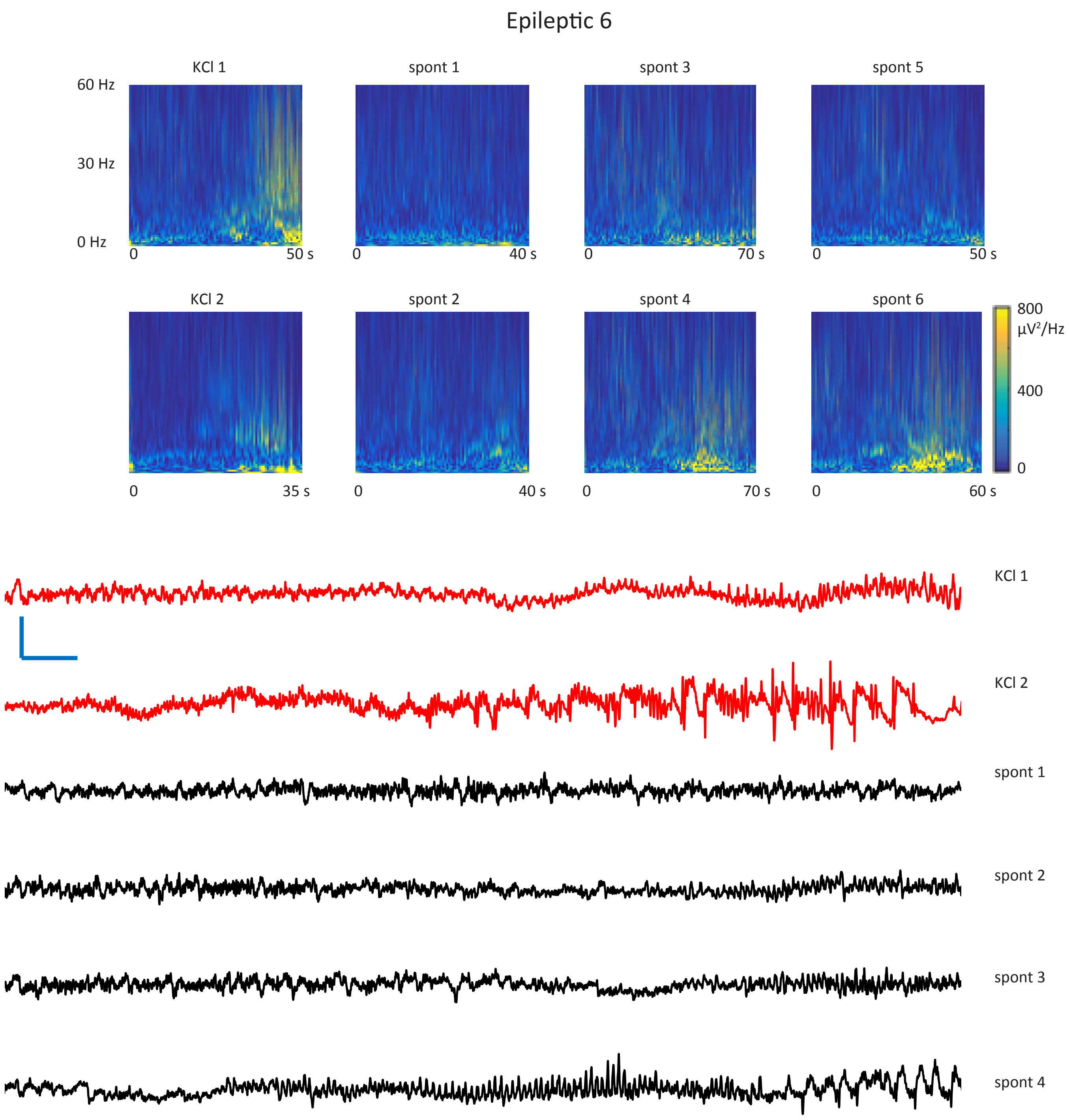

Morlet plots (top) and raw data (bottom) in Epileptic 6. KCl 1 is similar to several of the spontaneous seizures, but KCl 2 is quite different. This animal's induced seizures were deemed different by the SVM analysis in Fig. 4. Scale bars: 0.5 mV, 1 s

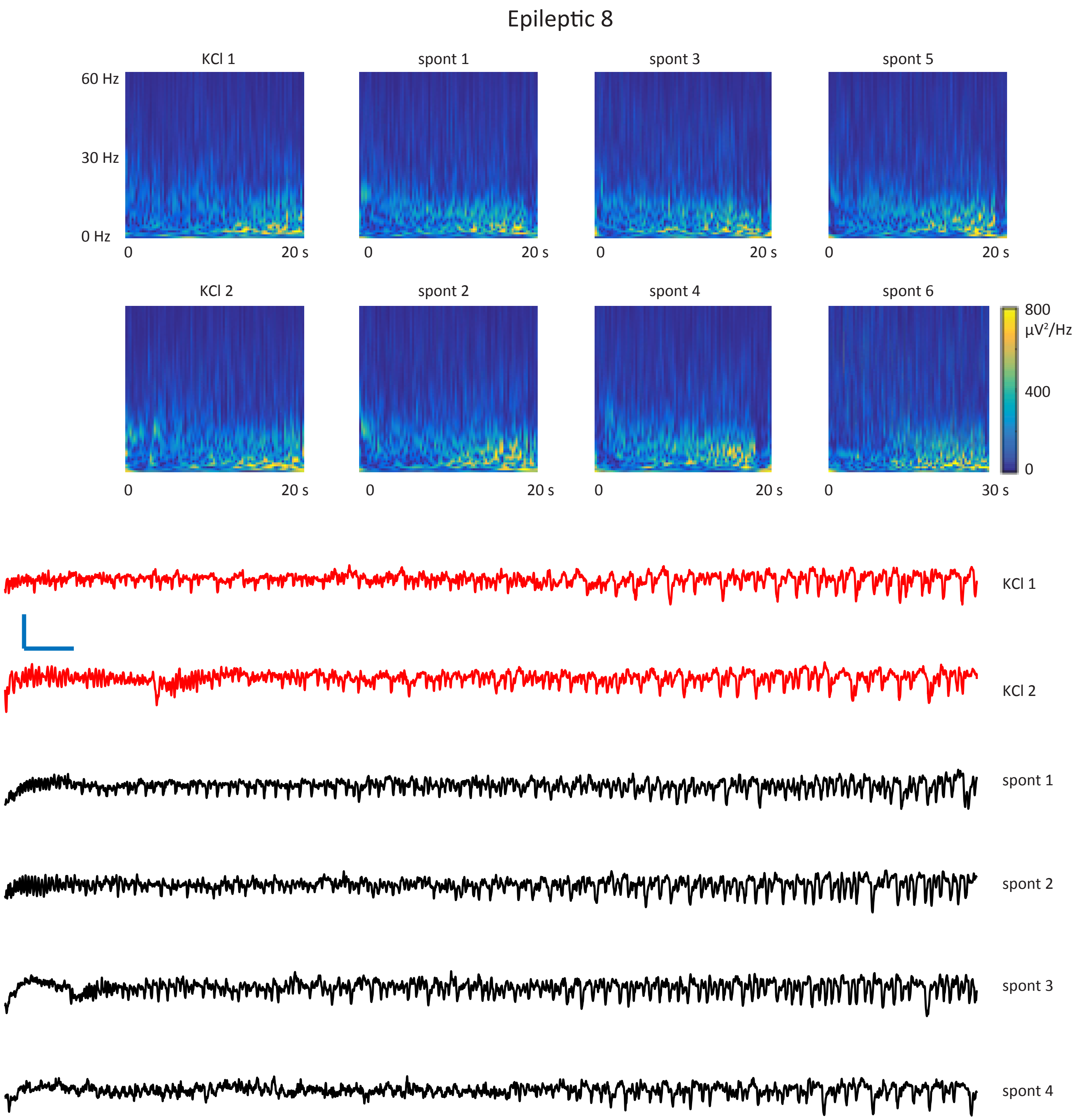

Morlet plots (top) and raw data (bottom) in Epileptic 8. All seizures have essentially identical appearance. Scale bars: 0.5 mV, 1 s

Epileptic 9

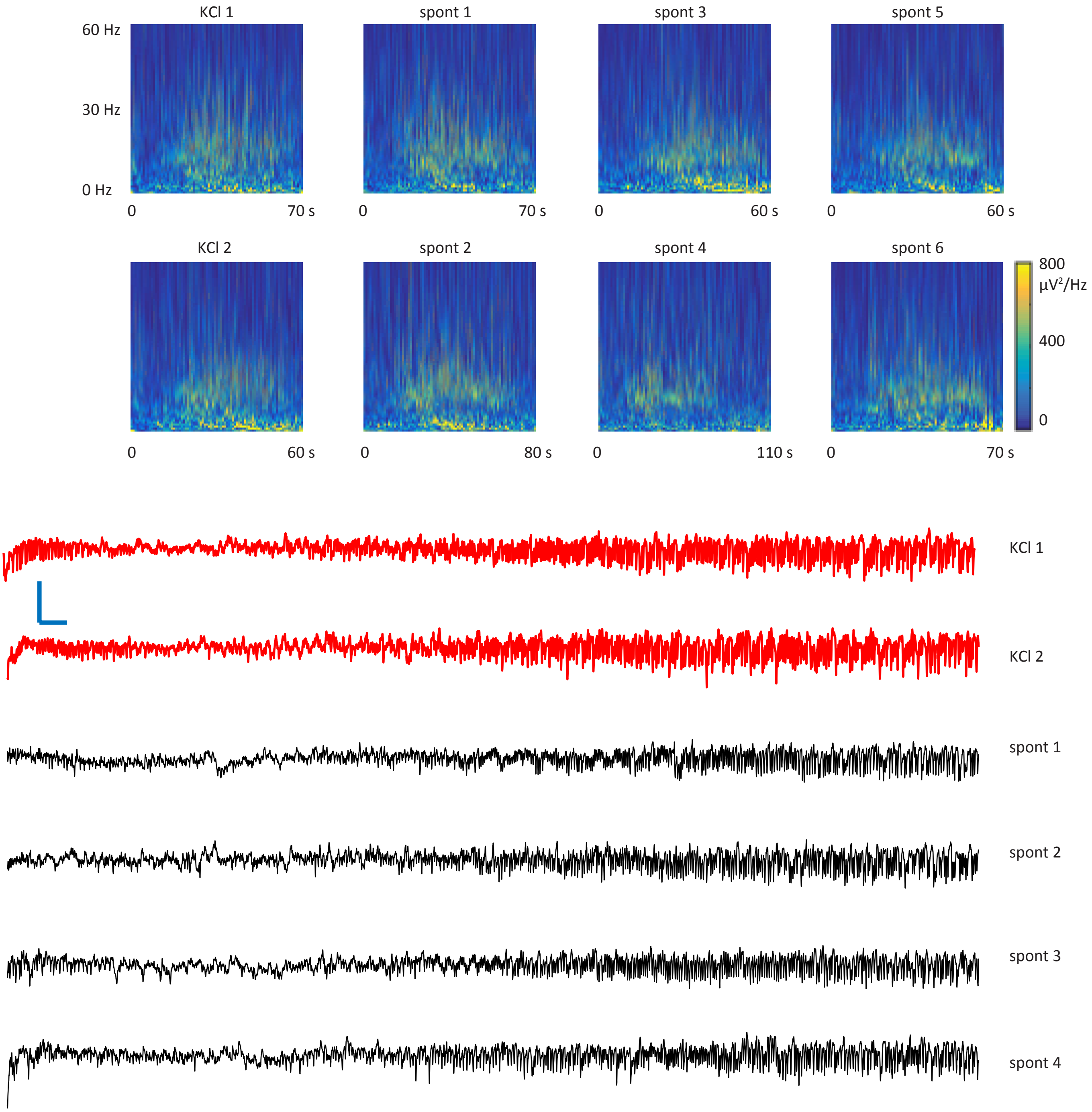

Morlet plots (top) and raw data (bottom) in Epileptic 9. All seizures have essentially identical appearance. Scale bars: 0.5 mV, 1 s

Epileptic 10

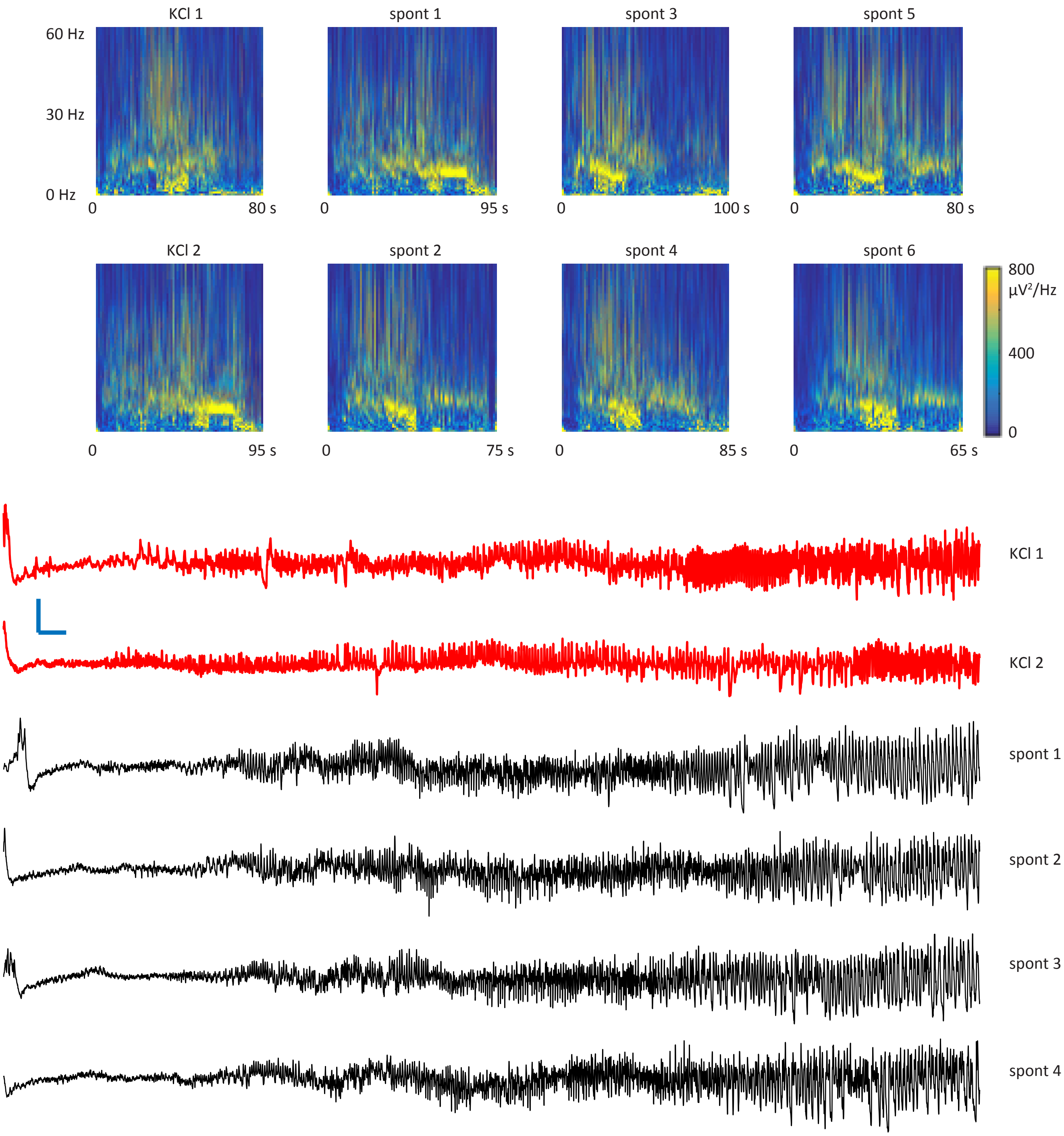

Morlet plots (top) and raw data (bottom) in Epileptic 10. All seizures accelerate to high frequency activity at about 30 sec and have similar long durations. Morlet plots have a distinct appearance that is present in all induced and spontaneous seizures. Scale bars: 0.5 mV, 1 s

Epileptic 11

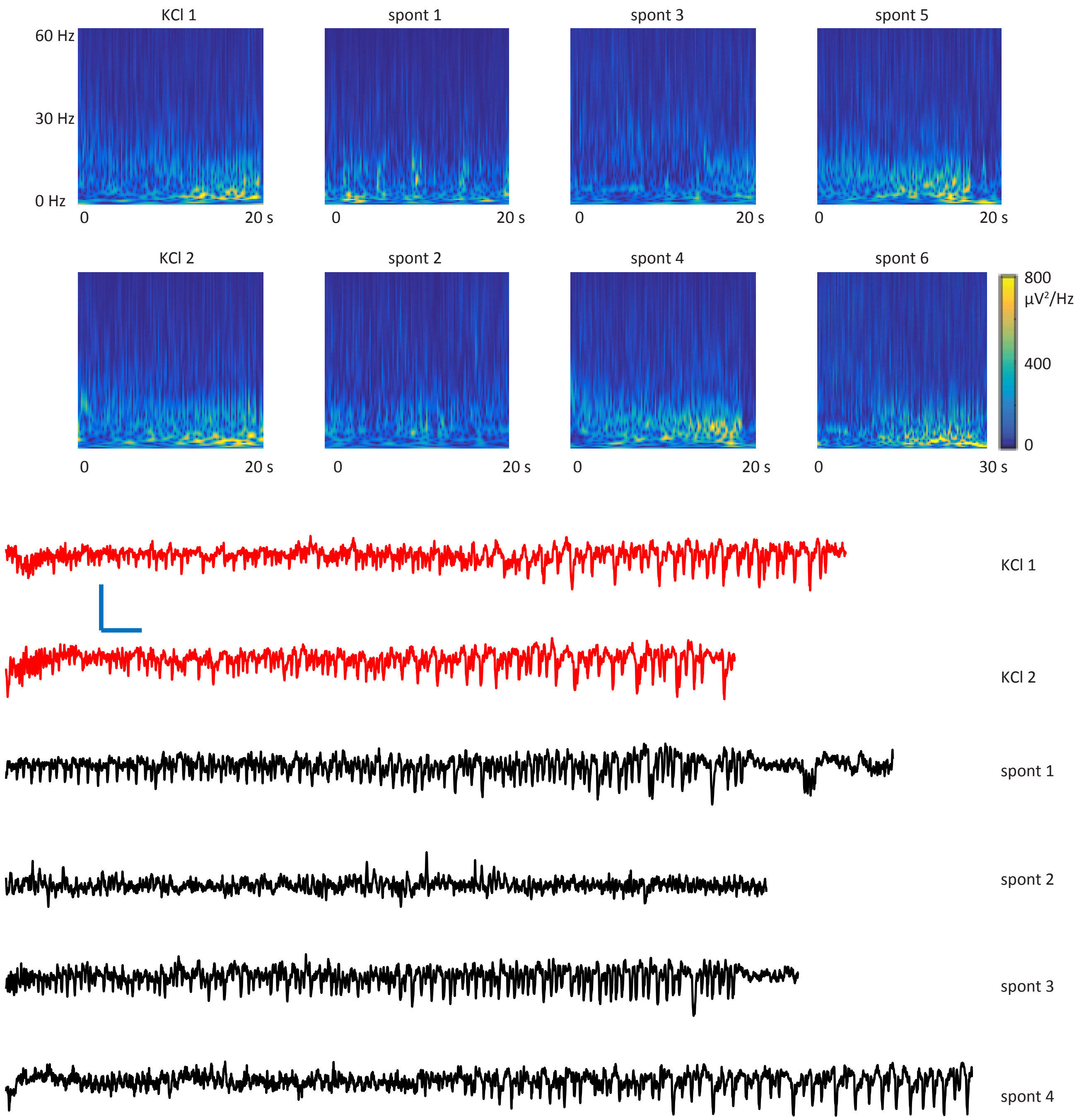

Morlet plots (top) and raw data (bottom) in Epileptic 11. The primary variability was within the spontaneous seizures, e.g. Spont 2 with different spiking morphology. The induced seizures were very similar to most of the spontaneous ones. Scale bars: 0.5 mV, 1 s

Epileptic 12

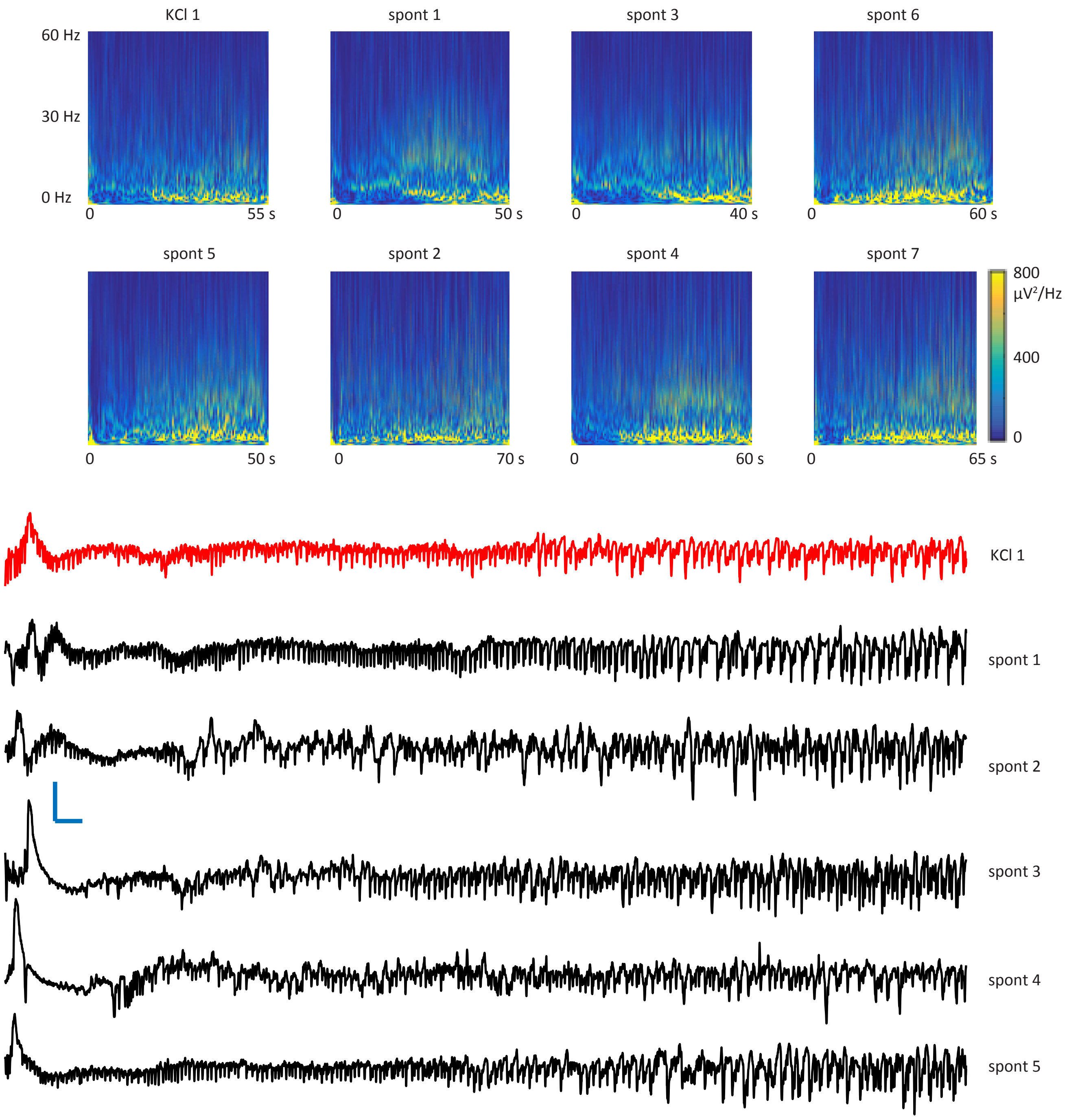

Morlet plots (top) and raw data (bottom) in Epileptic 12. The one induced seizure has the same distinctive waveform on initiation, as well as the fast downgoing spikes. Scale bars: 0.5 mV, 1 s

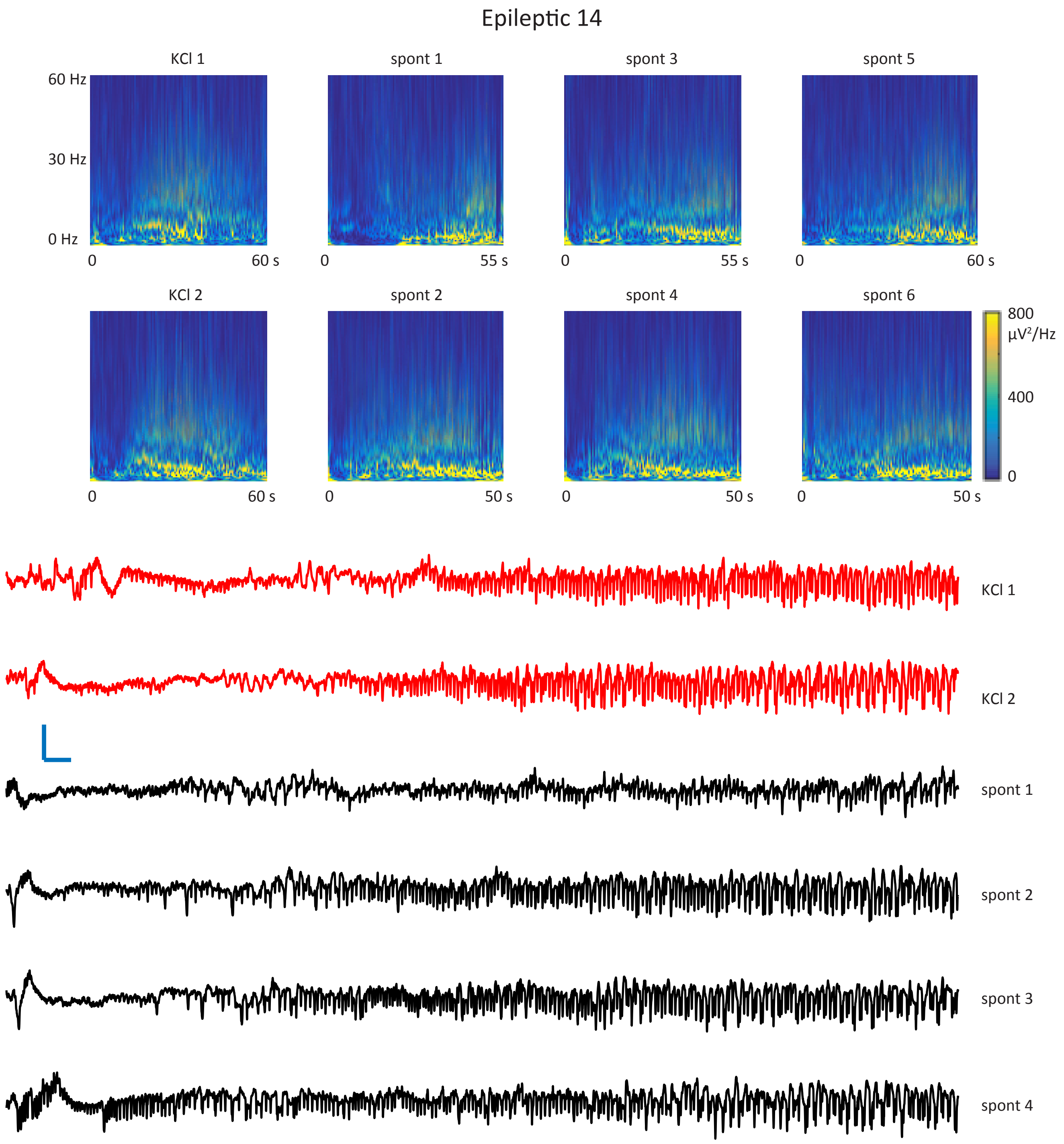

Morlet plots (top) and raw data (bottom) in Epileptic 14. KCl-induced seizures have the same initial deflection followed by low voltage fast, leading into larger amplitude firing.
Scale bars: 0.5 mV, 1 s

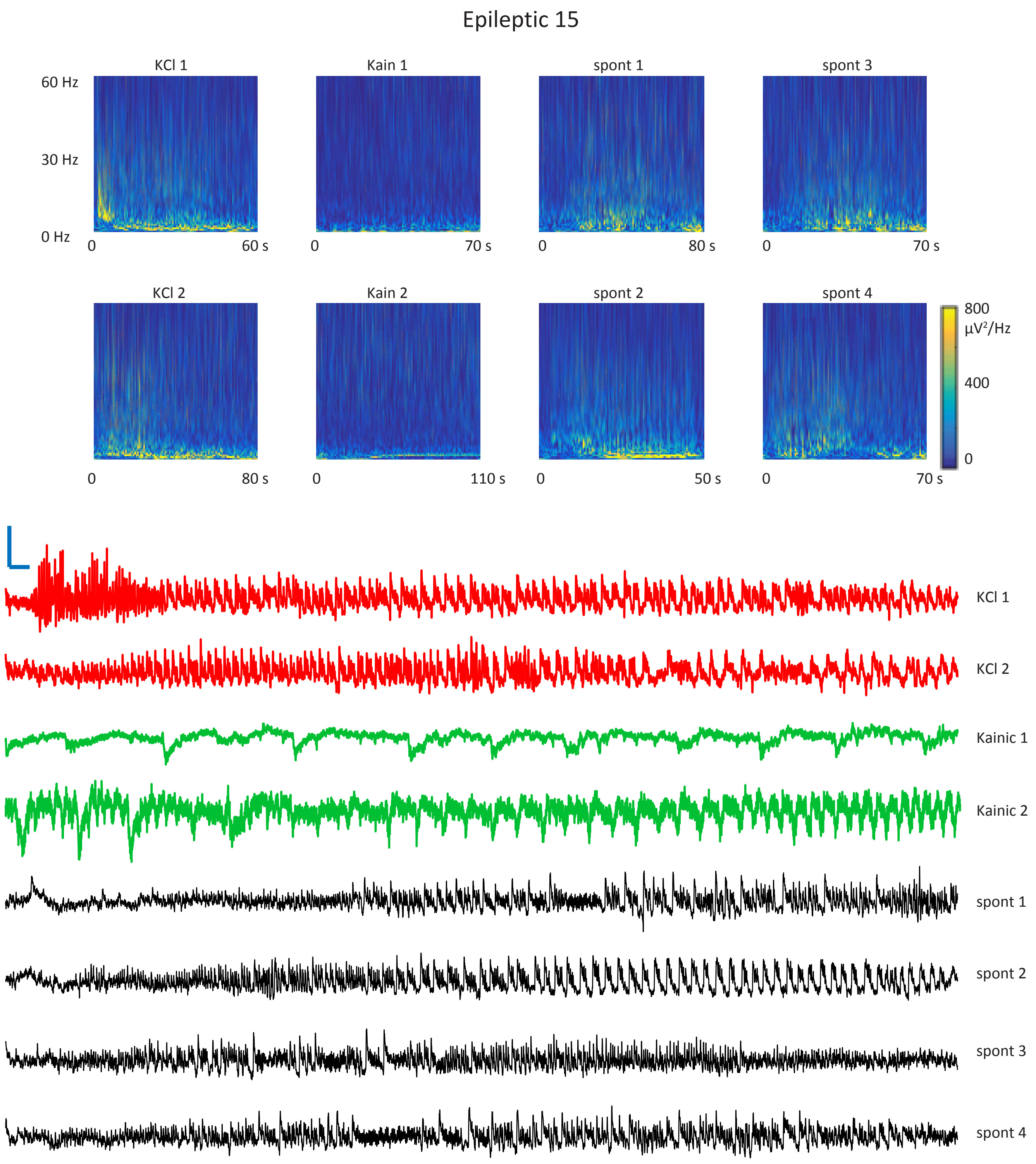

Morlet plots (top) and raw data (bottom) in Epileptic 15. KCl-induced seizures (red) are similar to some of the spontaneous seizures (e.g. 'spont 2'), which show some variability. The kainic acid-induced seizures (via direct injection to the hippocampus) have a distinct appearance: prolonged seizures, less high frequency activity, and reversed polarity (green). Three seizures were induced in response to a single kainic acid injection. Scale bars: 0.5 mV, 1 s

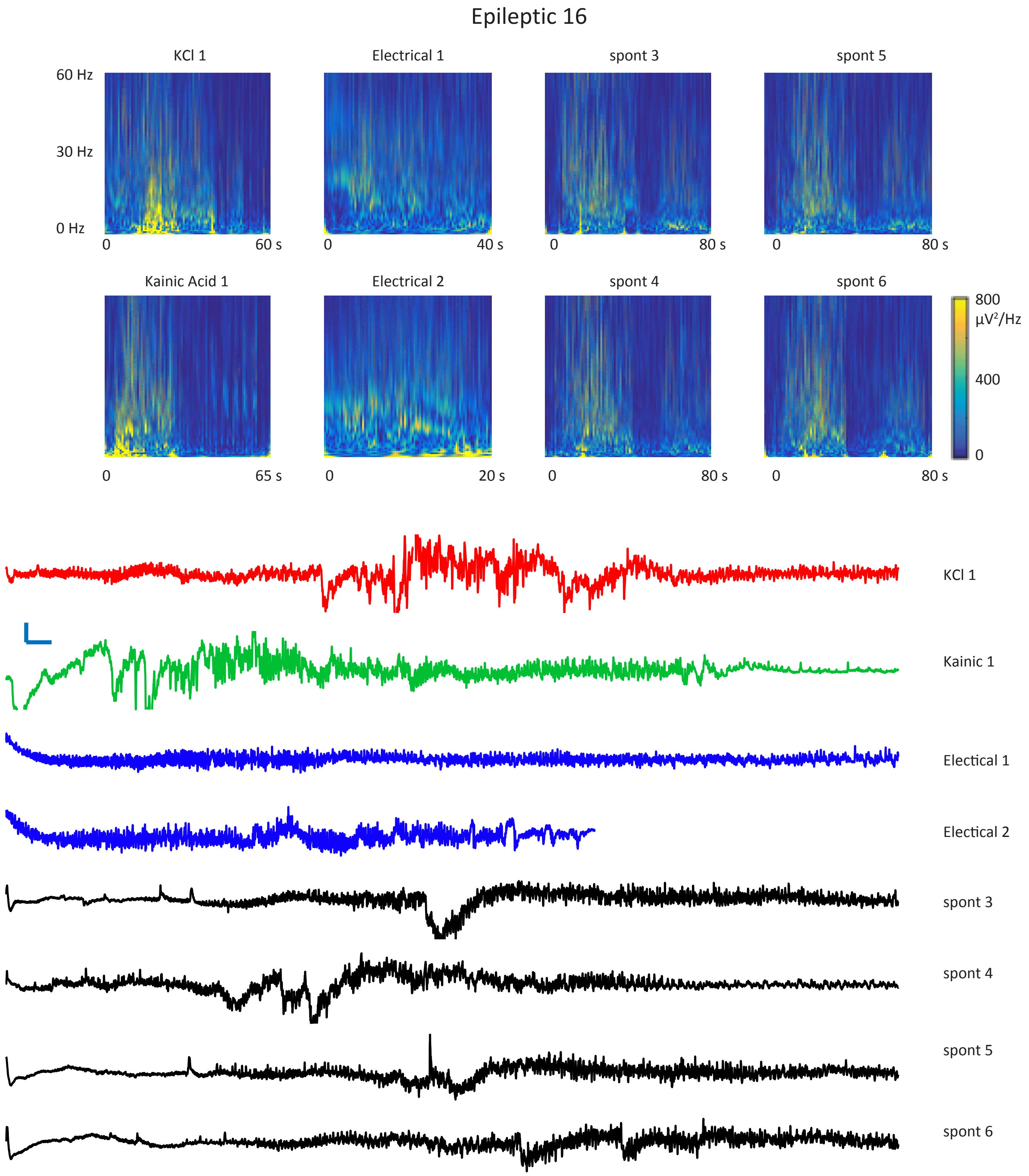

Morlet plots (top) and raw data (bottom) in Epileptic 16. The spontaneous seizures had a distinct appearance that was somewhat similar to the KCl- (red) and kainic acid- (green) induced seizures. The electrical stimulation-induced seizures (blue) consisted only of low voltage fast activity and were much shorter. Scale bars: 0.5 mV, 1 s
